# Clinical implications of microbial biofilms in chronic rhinosinusitis and orbital cellulitis

**DOI:** 10.1186/s12886-016-0340-z

**Published:** 2016-09-21

**Authors:** Niranjan Nayak, Gita Satpathy, Sujata Prasad, Alok Thakar, Mahesh Chandra, TC Nag

**Affiliations:** 1 Department of Ocular Microbiology, Dr Rajendra Prasad Centre for Ophthalmic Sciences, All India Institute of Medical Sciences, Ansari Nagar, New Delhi 110029 India; 2 Division of Oculopasty, Dr Rajendra Prasad Centre for Ophthalmic Sciences, All India Institute of Medical Sciences, Ansari Nagar, New Delhi 110029 India; 3 Department of Anatomy, All India Institute of Medical Sciences, Ansari Nagar, New Delhi 110029 India; 4 Department of Otorhinolaryngology, All India Institute of Medical Sciences, Ansari Nagar, New Delhi 110029 India; 5 Department of Microbiology, Manipal College of Medical Sciences, Pokhara, Nepal

**Keywords:** Biofilm, Amphotericin B, Obital Cellulitis, Chronic Rhinosinusitis

## Abstract

**Background:**

Discovery of sessile mode of microbial existence (Biofilm state) focussed much interest, during the recent years, on the study of biofilms in many recurring and chronic infections. However, the exact role of microbial biofilms in chronic rhinosinusitis and orbital cellulitis were not elucidated earlier. The purpose of the present study was to look for the adherent property and biofilm producing ability of the clinical isolates in chronic rhinosinusitis and orbital cellulitis, and to look for the effects of antimicrobial agents on these biofilms by colorimetric assay and ultrastructural analysis.

**Methods:**

Organisms were isolated and identified from various clinical samples in patients with chronic sinusitis and orbital cellulitis. Antimicrobial sensitivity testing was carried out by the standard protocol. Biofilms were developed; quantified and antimicrobial drug perfusion through the biofilm model was evaluated by the earlier devised procedure. Electronmicroscopic study of the biofilm was performed by the recommended technique.

**Results:**

Of the total of 70 clinical samples processed, 48 i.e. 68.5 % grew bacteria and 13 i.e.(18.6 %) fungi*. Staphylococcus aureus* (20), *S epidermidis* (16) and *Pseudomonas aeruginosa* (6) accounted for the majority of the bacterial isolates. *Aspergillus flavus* (8), however was the commonest amongst the fungi. A total of 40 bacteria and 8 fungi could be tested for biofilm production. Eighteen (45 %) of the 40 bacterial isolates and 4(50 %) out of the 8 *A flavus* isolates were found to be biofilm producers. In vitro adherence testing revealed that majority i.e. 16 (88.8 %) of the 18 biofilm positive bacteria were adherent to artificial surfaces. Antimicrobial drug perfusion through the biofilm model was poor. Antimicrobial treatment was totally ineffective against strong biofilm producers, whose electron microscopic picture was quite similar to that observed for biofilm producers without any antimicrobial pre-treatment.

**Conclusions:**

Filamentous fungi, like bacteria were capable of forming biofilms, which could be one of the important virulence factors in determining the pathogenic potential of these organisms in causing chronic rhinosinusitis and orbital cellulitis.

## Background

Biofims are highly organised microbial communities enclosed within a self-produced extra cellular polymeric matrix [1]. Biofilms, in an vivo situation, are highly recalcitrant to the defence mechanism of the body as well as to antimicrobial therapy [2]. There are scanty reports on the role of biofilms in acute fulminant infections such as orbital cellulitis and severe form of chronic rhinosinusitis(CRS).

Evidences in favour of biofilm producing organisms contributing towards the pathogenesis of osteomyelitis, and infections of various other cavities such as the pouch of Douglas and dental root canals were documented earlier [3]. However, the precise link between biofilm production and development of sino-nasal and orbital infections is still elusive*,* excepting that limited studies in the recent past described the role of bacterial biofilms alone, and that too in animal models of sinusitis [4].

Thus it is important to extrapolate the behaviour of the common bacterial and fungal pathogens causing sino-nasal and orbital infections, looking towards their biofilm forming abilities and other phenotypic characteristics, so that new perspectives on the pathogenesis and therapeutic modalities of such chronic as well as fulminant life threatening conditions, could be proposed.

This study was, therefore, planned with the aim of exploring the potential of the bacterial and fungal agents causing orbital cellulitis and CRS to form biofilms, and secondly to find out, if such biofilms could circumvent the effect of antibacterial and antifungal agents by restricting their entry through the sessile biofilm architecture in an in vitro model, designed in our laboratory.

## Methods

The study was conducted in the department of Ocular Microbiology of the All India Institute of Medical Sciences, New Delhi, India after obtaining the informed consent from the patients and the ethical clearance from the Institute’s ethical committee.

### Isolation and identification of pathogens from clinical specimens

#### Specimen collection

Aspirates in case of orbital cellulitis were collected by means of sterile syringe and needle with all aseptic precautions. Surrounding healthy skin was disinfected before sample collection. Pus, wound and/or sinus discharge were collected by rubbing the area with sterile cotton tipped swabs. However, if material was found to be insufficient, the wound/sinus was squeezed and the purulent exudates were collected. Thick brownish nasal discharge, when present was obtained with the help of sterile cotton swab. Eschar from the hard palate, if any, was collected by vigorously rubbing the swab deep in the perforated area of the hard palate. In lone cases of sinusitis nasal lavages/sinus wash materials were collected and immediately sent to the laboratory in sterile containers for further processing.

A total of 70 samples were obtained which comprised of 64 specimens of pus/aspirate/wound swab from cases of orbital cellulitis with/without clinical and/or radiologic evidence of sinus involvement; and six specimens (five sinus drains and one ethmoidal biopsy material) from cases having sinusitis alone.

#### Processing of specimens

All samples were processed in the laboratory following standard techniques. Direct microscopy on 10 % KOH wet mount was performed for demonstration of fungal elements in tissue. All specimens were cultured by inoculation onto Sabouraud’s dextrose agar (SDA) for growth of fungi and conventional bacterial culture media for growth of bacteria. Fungal isolates were identified by lactophenol cotton blue wet mount, slide culture if needed in case of mycelial fungi, and by germ tube test, pseudohyphae formation on corn meal agar and biochemical tests (sugar fermentation and assimilation and nitrate assimilation tests) for yeast species [5]. Bacteria were identified by the recommended techniques by studying the colony morphology on blood agar, chocolate agar and Mac Conkey’s agar and by interpreting various biochemical test results [6].

### Testing for the phenotypic markers

#### Biofilm assay

##### In vitro adherence and biofilm formation by bacteria

The isolates were tested for their ability to adhere to artificial surface and to form biofilms by adopting the previously standardized techniques mentioned elsewhere [7].

### Development of bacterial biofilm model on polycarbonate membrane

**Fig. 1:**
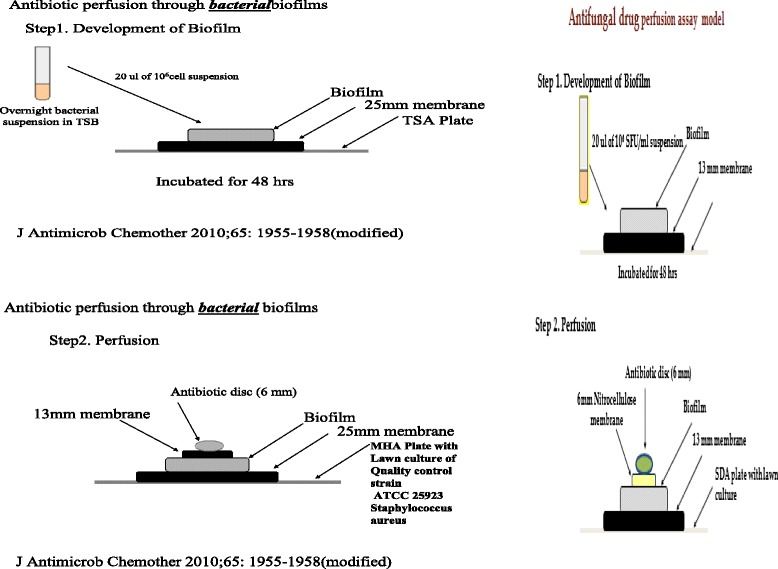
Quantitation of antimicrobial agents through Biofilm

Biofilms were developed on 25 mm black polycarbonate membrane as detailed previously [8], with minor modifications (Fig. 1). Antibiotic perfusion through the biofilm developed on this membrane was performed as depicted in step 2 of Fig. 1 [8].

#### Fungal biofilms

Growth conditions and standardization of conidial inoculum.

Fungi were grown on SDA at 37 °C for 72 h. Conidia were harvested by flooding the surface of the agar plates with 5 ml PBS (Oxoid) containing 0.025 % (v/v) Tween 20 and rocking gently. The conidial suspensions were recovered and dispensed into sterile glass containers. The conidia were counted using a Neubauer haemocytometer and adjusted to the required concentration in RPMI 1640 (Sigma) buffered to pH 7.0 with 0.165 M MOPS {3-(N-morpholino) propane sulfonic acid}. All procedures were carried out in a laminar flow cabinet as described previously [9].

### Biofilm formation

Fungal biofilms were formed on commercially available, pre-sterilized, polystyrene, flat-bottomed, 96-well micro-titre plates, according to the earlier described method [9, 10]. Briefly, 200 μl of a standardized cell suspension in MOPS-buffered RPMI 1640 were added to each well for selected time periods (4, 8, 12, 24 and 48 h), and incubated statically at 37 °C. A minimum of 12 replicates were performed for each experimental parameter, along with suitable controls. At each selected time point, the medium was aspirated and the biofilms were washed thoroughly three times with sterile PBS by repeated gentle pipetting to remove non-adherent cells.

### Quantification of antifungal agents through fungal biofilms

Antifungal activity was quantified using an XTT reduction assay, as described before [9]. Cellular viability was calculated as a function of metabolism, which was indicated by a change of colour from orange to pink depending on the relative viability of the filamentous population [9]. The metabolic activity of each phase of filamentous growth was quantified in a microtitre plate reader (FLUOstar OPTIMA, BMG Labtech, Buckinghamshire, UK) at 490 nm. The XTT absorbance value, after normalizing for back-ground absorbance levels, was used to assess the effectiveness of each antifungal agent relative to the unchallenged positive control. Sessile MICs (SMICs) were determined by comparing the intensity of colour change with the untreated control. Testing of each isolate was performed in triplicate.

### Ultrastructural analysis

Ultrastructural study was performed by electron microscopy of bacterial and fungal biofilms on polycarbonate surfaces and also on antibiotic treated biofilm after developing antibiotic biofilm model represented schematically vide Fig. 1. Biofilm, thus developed, was fixed with 5 % glutaraldehyde, treated with Osmium tetroxide (OSO_4_) and dehydrated with ethanol before being subjected to electron microscopic study [11].

### Antimicrobial sensitivity testing

The isolates were tested against antifungal agents according to the guidelines laid down by CLSI (M-38A, USA). Antibacterial sensitivity was performed by the recommended Kerby Bauer disc diffusion method [12].

### Development of biofilm model on polycarbonate membrane

For standardization, overnight bacterial culture in trypticase soy broth (TSB) was added to the black polycarbonate membrane (25 mm diameter) on trypticase soy agar (TSA) plate and incubated till 48 h at 37 °C for biofilm development. Perfusion of antibiotic through the biofilm was tested as per the protocol depicted in Fig. 1. The same technique was adopted for fungal biofilm development and for antifungal drug perfusion assay (Fig. 1).

## Results

A total of 70 samples were collected; 64 pus/aspirates, five sinus drains and one ethmoidal biopsy material. Of the 64 orbital cellulitis specimens, 46 (72 %) yielded bacteria and 9 (14 %) grew fungi. The 46 bacterial isolates comprised of 20, *Staphylococcus aures*; 16, *Staphylococcus epidermidis*, 4, *Pseudomonas aeruginosa,* 4 aerobic spore bearing organisms and one each of *Streptococcus pneumoniae* and *Klebsiella pneumoniae.* Out of the nine fungal apthogens obtained from orbital cellulitis cases, four were *Aspergillus flavus*, two *Alternaria* species and there was one each of *Curvularia, Fusarium* and *Candida albicans*. All the six samples from sinusitis cases were culture positive, showing growth of *Aspergillus flavus* in four and *Pseudomonas aeruginosa* in two. Overall, culture positivity for bacteria was found in 68.5 % (48 of 70) of the cases and for fungi in 18.6 % (13 of 70) cases.

Forty out of the total of 48 bacterial isolates were tested for their biofilm production. These 40 comprised of 20 isolates of *Staphylococcus aureus,* 16 *Staphylococcus epidermidis* and 4 *Pseudomonas aeruginosa* (two each from sinus and sino-orbital sites). All eight *Aspergillus* isolates were also tested for biofilm production. It was noted that 18 (45 %) of the 40 bacteria were biofilm positive. Majority of the biofilm producing organisms (16/18 i.e. 88.8 %) were capable of adhering to polysterene surfaces as compared to only 7 (31.8 %) of the total of 22 non-biofilm producing organisms (Table 1). This difference was found to be statistically significant. Amongst the eight Aspergillus isolates,however, 4(50 %) were noted to be biofilm producers.

**Table 1:** Adherence properties of the 40 bacteria tested for biofilm production

Biofilm production	Adherent	Non-adherent	Total
Biofilm positive	16 (88.9 %)	2 (11.1 %)	18
Biofilm negative	7 (31.8 %)	15 (68.2 %)	22

χ ^2^ = 13.12; *p* <0.001

**Fig. 2:**
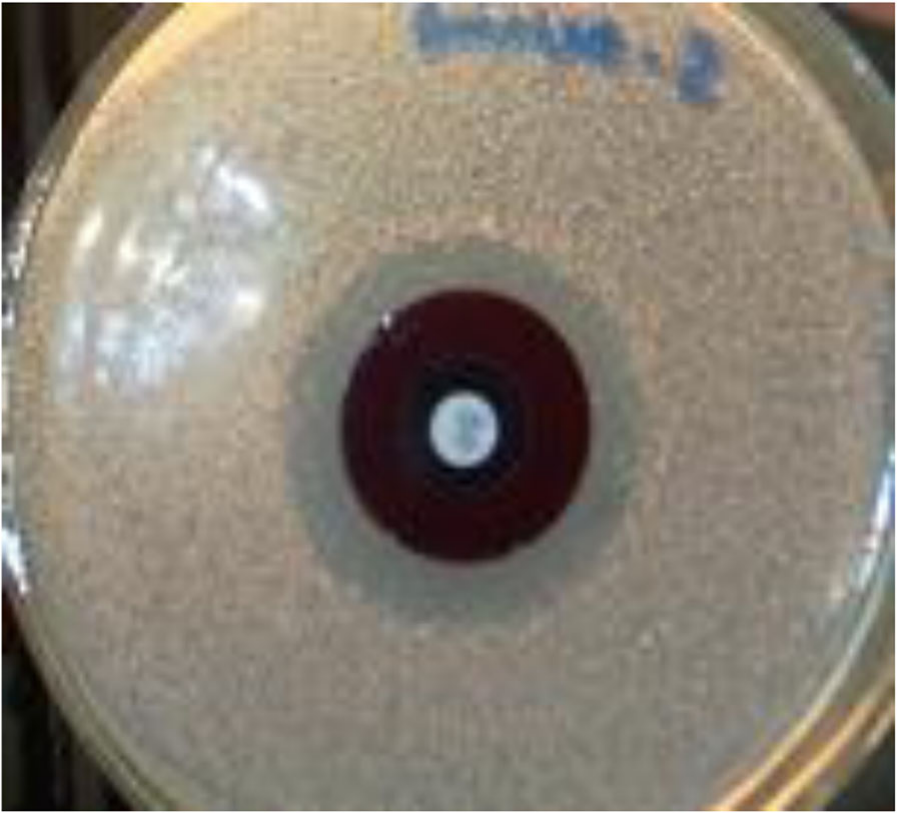
Control plate without biofilm which shows clear cut zone of inhibition beyond the black poly carbonate membrane. The lawn culture on the plate is that of a clinical isolate of *Staphylococcus epidermidis* from a case of orbital cellulitis. Antibiotic disc is placed in the centre

**Fig. 3:**
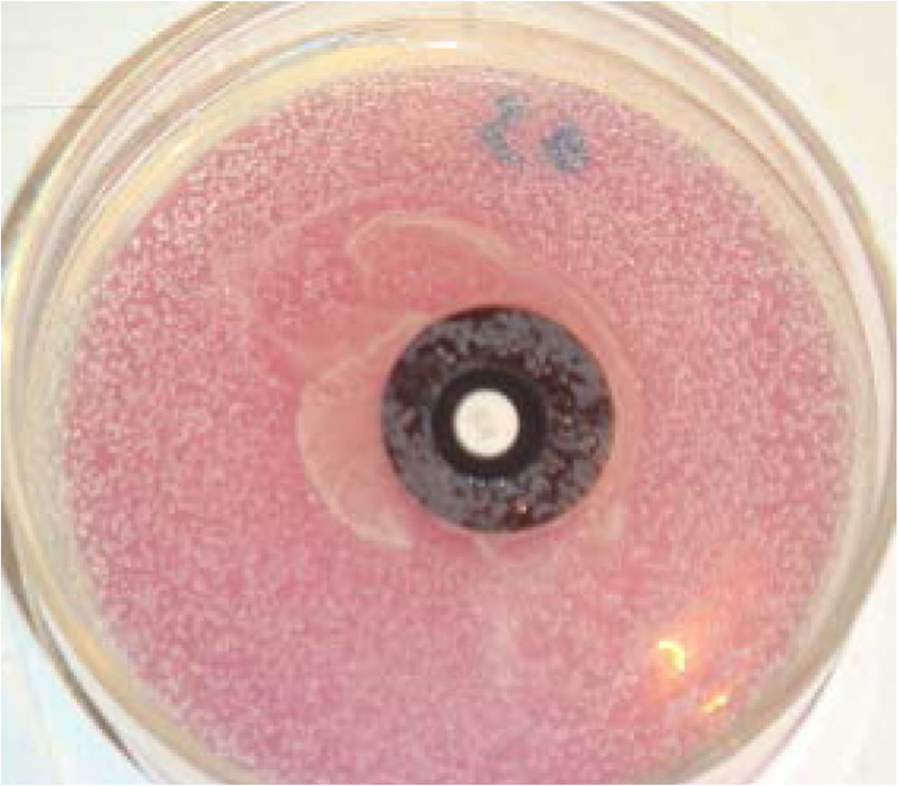
Shows no zone of inhibition around the black poly carbonate with the biofilm developed on it. Antibiotic disc is placed in the centre

Figure 2 depicts the results of the standardization of antibiotic perfusion through the biofilm model, as described in the methods. The standardization protocol yielded optimum results showing clear zone of inhibition beyond the black polycarbonate membrane when there was no biofilm over the membrane. Similarly Fig. 3 documents a representative of the strain producing biofilm that did not allow any perfusion of antibiotics, and therefore, indicating no zone of inhibition surrounding the polycabonate membrane. Subsequently all clinical isolates were tested for antibiotic perfusion through their biofilms developed in accordance with this standardized technique.

**Fig. 4:**
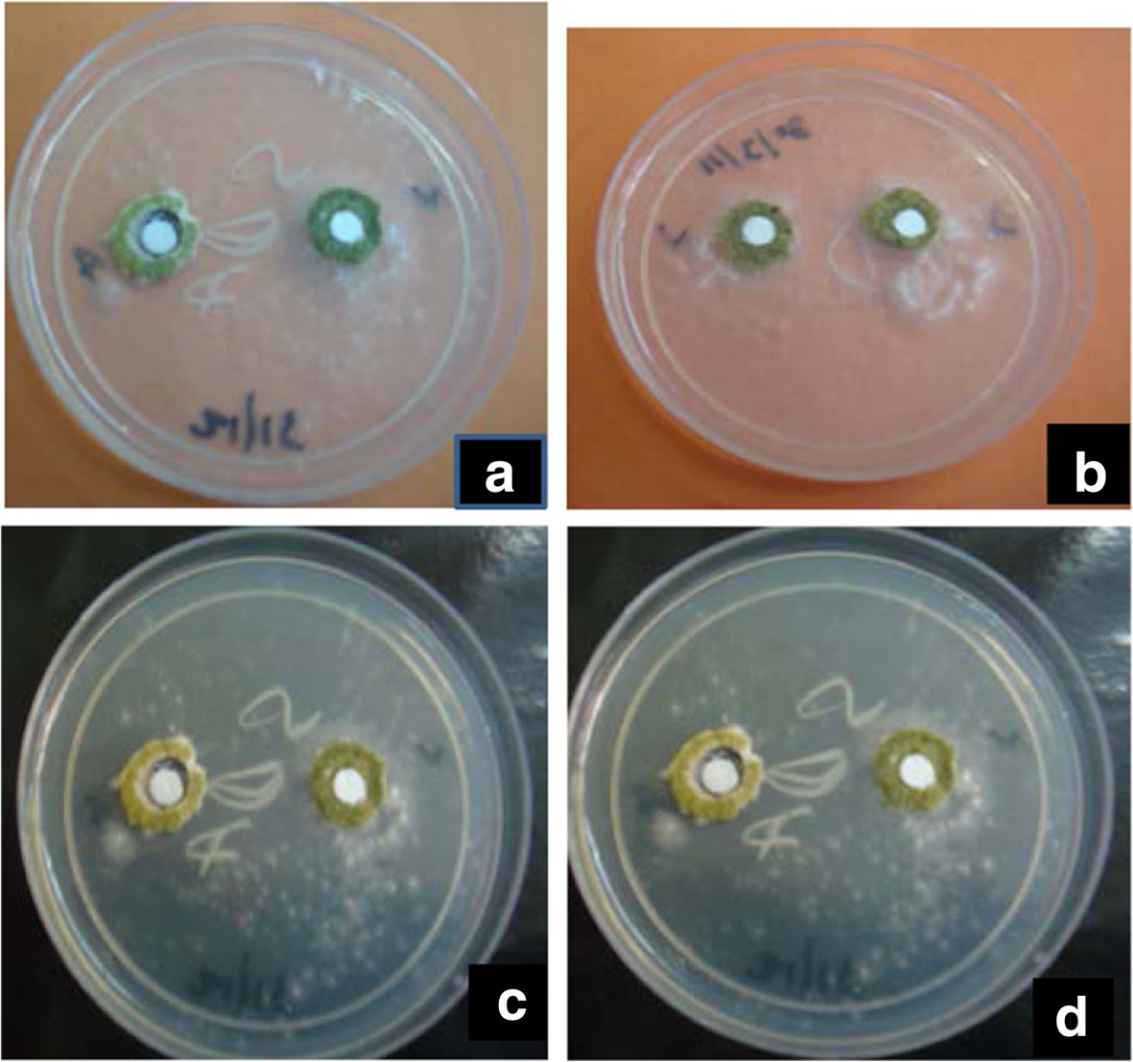
Antifungal drug perfusion through fungal biofilm on black polycarbonate membrane showing moderate zone of inhibition around the left most discs of the lower two plates i.e. panels **c** and **d**, whereas in rest of the six sectors (two each in panels **a** and **b**; one each in panels **c** and **d**), there is no zone of inhibition suggesting that antifungal agent did not perfuse through the biofilm developed on the polycarbonate membrane. All the eight organisms tested were Aspergillus flavus isolates from orbital pus, sinus drain and biopsy materials

**Fig. 5:**
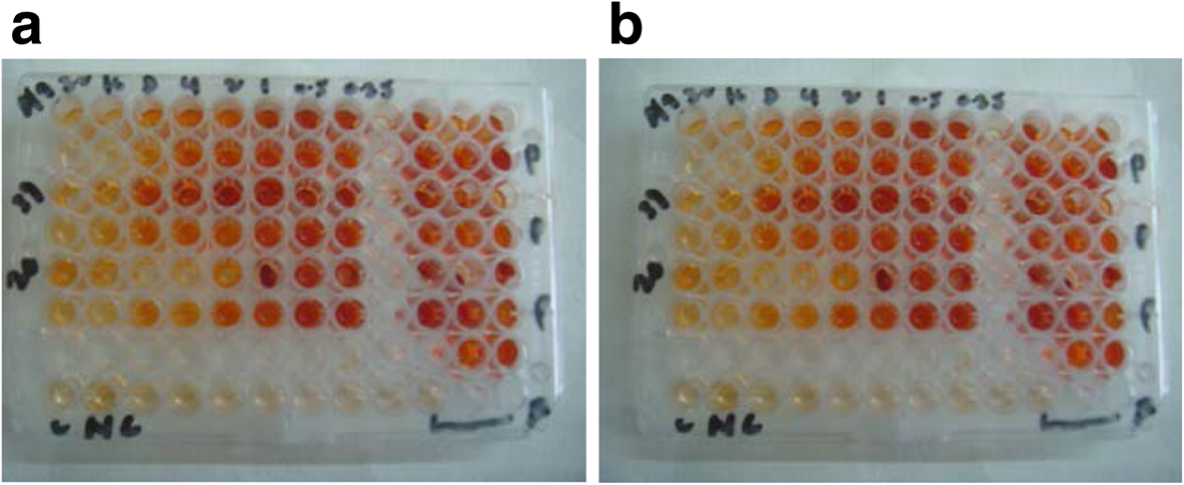
XTT reduction assay to show resistance patterns of fungal biofilm to amphotericin B. Panels show the MIC results of six Aspergillus biofilms (rows 1–6) in duplicate (Panel **a** and Panel **b**) showing amphotericin B MICs of 4,4,8,4,1 and 2 mg/L respectively in the rows 1, 2, 3, 4, 5 and 6. Strains in the row 3 with MIC value of 8 mg/L was a strong biofilm producing organisms and those in the rows 1,2 and 4 were weak biofilm producers as shown by the SEM study. Wells in the right most panels show positive controls without antifungal agent and those in the row below (labelled as “NC”) show the negative controls i.e. dye alone without any organisms exhibiting no reduction of the dye and thus no color intensity

Figure 4 demonstrates the antifungal drug (amphotericin B) perfusion through *Aspergillus* biofilms developed on the black polycarbonate membranes. Panels A through D show SDA plates with spore inoculum lawn culture of all the eight clinical isolates of *A flavus* (two isolates per plate), along with the biofilm model demonstrating either inhibition or no inhibition of fungal growth. It was interesting to note that only two (left most sectors of plates shown in panels C and D) out of the eight fungi exhibited moderate zones of inhibition surrounding the disc, suggesting thereby that the rest six isolates showing no inhibition zone were biofilm producers. This finding was further authenticated by the results of the XTT reduction assay (Fig. 5) which demonstrated that four (upper four rows of either of the microtiter plates) of those six isolates (Fig. 4) that prevented amphotericin B perfusion through their biofilms, had shown high MIC values (between 4 and 8 mg/L). The above findings bear important clinical implications that *A flavus* capable of forming biofilm in vivo in diseased conditions, would be recalcitrant to antifungal medication, because cells in the interior of the biofilm would be least exposed to the drug owing to inadequate drug perfusion through the biofilm matrix. The absorbance values of the respective wells of the XTT reduction assay have been shown in Table 2 that exhibits increase in the absorbance values with relative decrease in drug concentration.

**Table 2:** XTT Reduction assay results

Drug concentration in μg per ml										
Lab. No	32	16	8	4	2	1 μ	0.5	0.25	Positive control	Negative control
39/12/9	2.307	3.011	2.894	2.793	3.372	3.325	3.325	3.306	3.316	0.143
20/13/9	1.876	2.002	1.997	2.297	2.833	2.176	2.961	3.506	3.522	0.167
29/3/9	1.604	1.632	1.656	1.788	1.823	2.772	2.827	2.884	3.513	0.18
30/3/11	1.337	2.421	2.557	2.697	2.697	2.971	2.986	2.999	2.985	0.199
16/5/6	0.941	1.14	1.477	1.419	1.563	1.596	2.247	2.564	OVRFLW	0.047
19/15/12	1.499	1.499	1.512	1.516	2.555	2.794	2.819	3.436	3.538	0.098
21/15/12	0.941	1.14	1.477	1.419	1.563	1.596	2.247	2.564	OVEFLW	0.1
12/23/2011	1.559	1.776	2.667	3.14	3.241	3.256	3.312	3.323	3.113	0.145

**Fig. 6:**
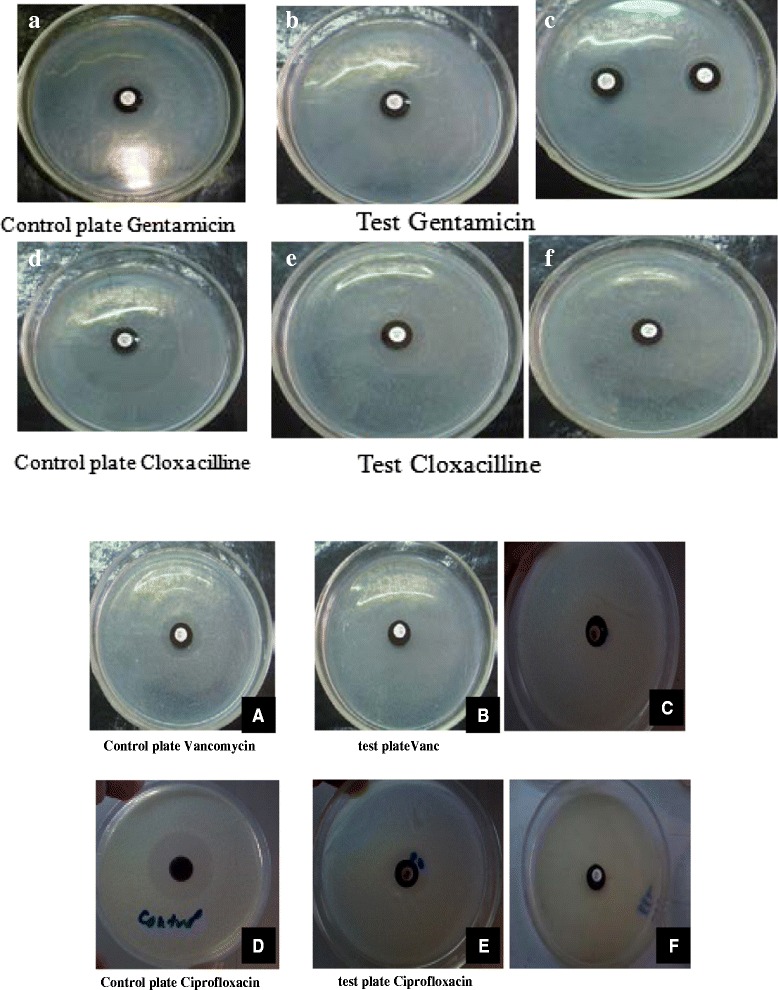
Shows antibiotic perfusion through various biofilm models developed on black polycarbonate membranes as described in the methods. Panels **a**, **d** and **A**, **D** above represent the controls without any biofilm on the polycarbonate membranes, showing clear zones of inhibition of bacterial culture tested against gentamicin, cloxacillin,vancomycin and ciprofloxacin respectively. Panels **b**, **c**, **e**, **f** and **B**, **C**, **E**, **F** represent models in which biofilms were developed on the membranes. Whereas panels **b**, **e** and **E** show clear zones of inhibition of bacteria surrounding the biofilm, other panels like **c**, **f**, **B**, **C** and **F** show either no zone or very minimal zone. Antibiotics tested on each model have been named below the individual panels

Figure 6 represents antibiotic perfusion assay through polycarbonate membrane with or without bacterial biofilms as described in the methods. Panels a, d, A and D are the controls without any biofilm on the polycarbonate membranes, showing clear zones of inhibition of *S epidermidis* lawn culture tested against gentamicin, cloxacillin, vancomycin and ciprofloxacin respectively. Panels b, c, e, f and B, C, E, F represent models in which biofilms were developed on the membranes. Whereas panels b,e and E show clear zones of inhibition of bacteria surrounding the biofilm, other panels like c, f, B, C and F show either no zone or very minimal zone. Antibiotics tested on each model have been named below the individual panels.

**Fig. 7:**
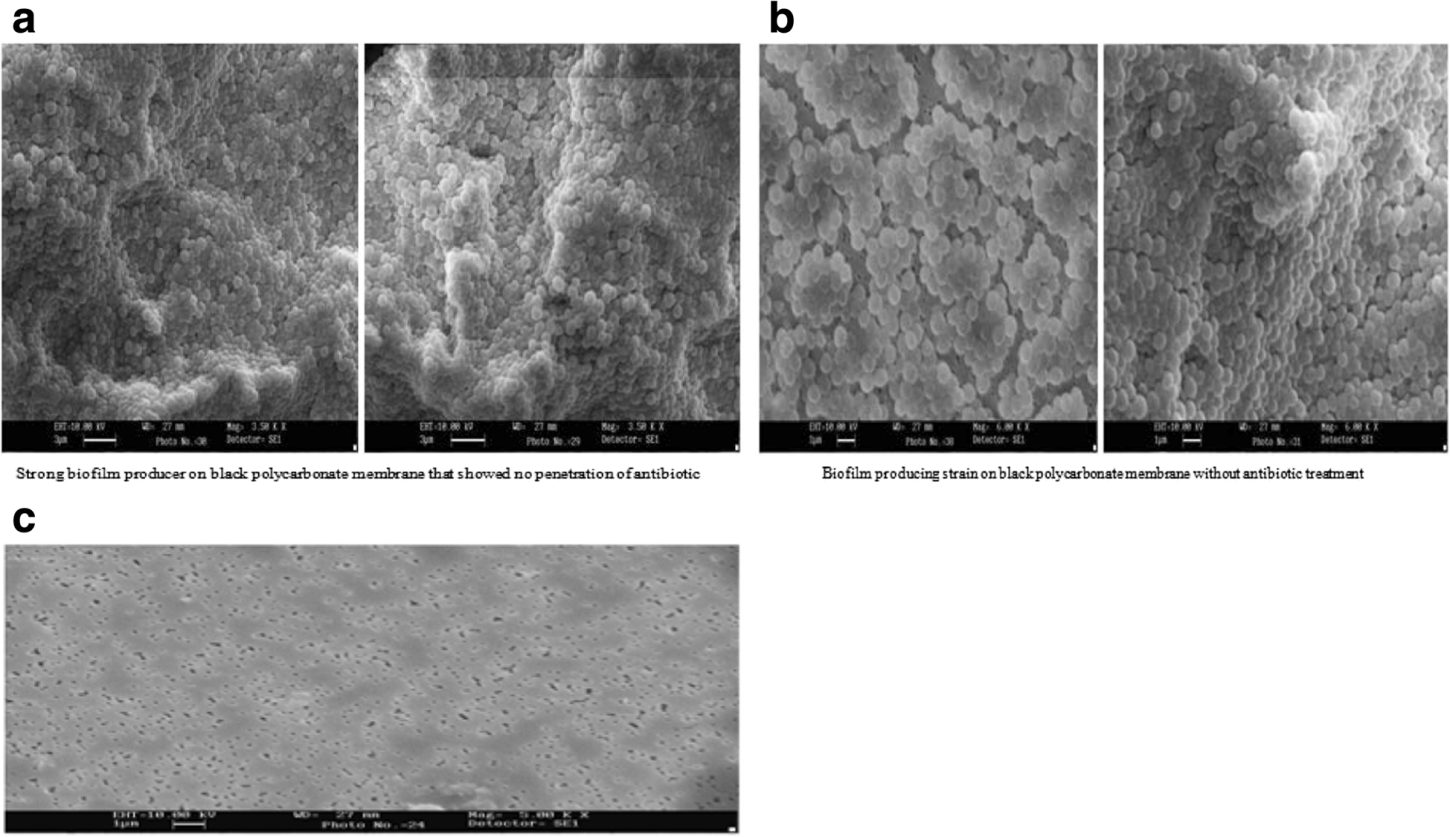
Panel **a** showing the SEM picture of strong biofilm producing bacteria on the black polycarbonate membrane that did not allow perfusion of antibiotics, which is quite comparable in density to another strong biofilm without any antibiotic treatment (Panel **b**). SEM picture of polycarbonate membrane alone has been depicted in panel **c** for comparison

It was quite evident from the above mentioned observations that zones of inhibition due to antibiotic perfusion across the biofilms was substantially less (or even no zone at all) as compared to those produced by antibiotics perfusing through the membrane alone without any biofilm overlying it. It will be noteworthy to mention here that bacterial isolates showing either no zone or very minimal zone of inhibition in the panels c, f, B, C and F were strong biofilm producers. These strong biofilms elaborated by the aforementioned five strains (in the panels c, f, B, C, F) exhibited thick closely packed cells framed in a sessile architecture, intimately attached to one another as evidenced by the SEM analysis (Fig. 7 panels a and b). Interestingly, there was not much variation in the population density of cells of strong biofilm producers whether they were treated or untreated with antibiotics (Fig. 7, panel a vs. b). This suggested that strong biofilm producing organisms resisted killing by antimicrobial agents.

## Discussion

Recent recognition of the ubiquitous nature of microbial biofilms has generated much interest among the scientific community in studying a number of infectious disease processes from a biofilm perspective. The riddle of biofilm is due to its sessile mode of growth giving rise to chronicity of infection and persistence of the organism inside the core of the biofilm resulting in recalcitrance towards antimicrobial chemotherapy [13]. Ventilator associated pneumonias, prosthetic valve endocarditis, periodontitis, bacterial keratitis, central venous catheter related sepsis, late onset endophthalmitis following intra-ocular lens implantation, prosthetic joint and other orthopedic implant related infections and chronic lung infections in cystic fibrosis are some of the examples of diseases that are generally encountered to be associated with microbial biofilms [13, 14]. These infections share some common characteristics even though the microbial causes and sites of infection in the host, show profound variation. The most important implication of this process is that micro-organisms in biofilms evade host defences and withstand antimicrobial pressure.

As emphasized above, microbial biofilms having a survival advantage due to evasion of host immunity and unresponsiveness to antimicrobial therapy, can give rise to serious infections, especially among ICU patients implanted with indwelling medical devices. These infections do not usually resolve unless the biofilm is dissolved or the implant is removed, which in many cases is not cost effective and sometimes, impracticable. Susceptibility tests using in vitro biofilm models have shown the survival of bacteria after treatment with antibiotics at concentrations hundred or even thousand times the minimum inhibitory concentration for the organism measured in a suspension culture i.e. in its planktonic state [8–10]. Administration of antimicrobial agents may suppress symptoms of infection by killing free floating organisms shed from the attached population, but fail to eradicate those buried in the interior of the biofilm. Soon after the antimicrobial chemotherapy is omitted, the same biofilm acts as a potential source for another spell of fresh infection leading to recurrence and chronicity. However, in spite of the aforementioned valuable information, studies on the importance and clinical implications of bacterial and fungal biofilms in CRS and orbital cellulitis were scanty.

In view of the aforementioned observations relating to biofilm and microbial virulence in various systemic infections, we tried to explore similar such occurrence in CRS and orbital cellulitis. It was justifiable to study these two disease entities together because these two are clinically very much inter-related so far as the pathogenesis of the later condition is concerned which often develops as a complication of the former (frequently ethmoidal sinusitis).

Cryer et al. [15] first suggested the presence of biofilms on the sinus mucosa of CRS patients. This short study of 16 CRS patients, who had failed to respond to both surgical and medical treatments, utilised scanning electron microscopy to analyse the specimens of sinus mucosa. In their experiments, the researchers found four specimens with a thicker coating than what is found in a normal muco-ciliary blanket. Further studies on the pathogenesis of various bacterial infections suggested that bacteria could adhere to solid surfaces and form a slimy, slippery coat. These, so called, bacterial biofilms were prevalent on most wet surfaces, and bacterial cells embedded inside biofilms were resistant to antimicrobial agents and the host immune defence mechanism [15].

Ferguson and Stolz [16] demonstrated the presence of bacterial biofilms in two of four patients with CRS. Also using scanning electron microscopy (SEM) and transmission electron microscopy (TEM), Sanclement et al. [17] noted a prevalence of biofilms in 80 % of the mucosal biopsies of CRS patients. Further electron microscopic research revealed the presence of biofilms in silastic stents removed from the frontal sinus recess after endoscopic sinus surgery [18]. We recently documented with the help of SEM that *S epidermidis* strains isolated from patients with invasive disease in the eye produced slime and had the potential to adhere [7, 11]. However, those were the isolates from infectious keratitis, and not from cases of orbital cellulitis. In yet another study, Imamura et al. [19] put forth the evidence of *Fusarium* and *Candida albicans* biofilms on soft contact lenses, though fungal biofilms were, then, not documented either in orbital cellulitis or in sinusitis.

Unlike the above mentioned studies on SEM and TEM claiming direct evidence of biofilms on tissue material [16–18], the present study, however, provided information that pathogens causing such infections were capable of forming biofilm in vitro (Fig. 6). Our results were in agreement with the findings shown by others [20]. Additionally, our study highlighted that strong biofilms on the polycarbonate membranes drastically resisted perfusion of antimicrobial agents through them, a finding much similar to those noted by others [8–10], who were of the view that perfusion of all antimicrobial agents was poor through the in vitro biofilm model. This perfusion model could mimic the in vivo situation when organisms producing biofilms either on indwelling medical devices or inside deep body cavities like sinuses and orbit, could give rise to chronic infections, unresponsive to antimicrobial treatment.

However, unlike the recent progresses made over the research on the effects of antibiotics on bacterial biofilm [8, 13, 14, 20], fewer studies are available, estimating the efficacy of antifungal drugs on fungal biofilms [21]. Recently, Mowat et al. [9] reported that all antifungal drugs tested, were at least 1000 times less effective in reducing the overall metabolic activity of 90 % of *Aspergillus fumigatus* biofilm cells as compared to their planktonic counterparts. Whereas Mowat et al. [9] tested the antifungal susceptibility of the organisms both in their sessile as well as in their planktonic forms; our study did not have any scope for comparison of the sessile MIC values with the planktonic MICs. This drawback of the present study could be attributed to the small sample size yielding only a few fungal pathogens. Nevertheless, the results of our study, could have definite indication that organisms inside the biofilm could be far less sensitive to antimicrobial agents than the planktonic cells, an observation much in agreement with that of Hasan et al. [22], who investigated *Candida* biofilms employing an in vitro XTT reduction assay, and concluded that biofilm formation was a stable character among clinical *Candida* isolates and such biofilms played an important role in persistence of infection. We assayed the biofilm producing abilities of *Aspergillus flavus* clinical isolates by adopting similar techniques as that of Hasan et al. [22], but our study, not only projected on the potential of the biofilm organisms to give rise to chronic persistent infection, but also on their recalcitrant nature towards commonly used antimicrobial agents in clinical practice.

The present study, thus, showed that majority of the adherent organisms were biofilm producers. These results are in concordance with those of other investigators [23], who observed that *S. epidermidis* recovered from clinical materials behaved differently from the commensal *S. epidermidis* not only by the presence of the *ica* A and the *ica* B adherence genes, but also by their tendency towards phase variation, attachment to polymer surfaces, and capabilities to produce biofilm. Frebourg et al. [24], recently, documented that significantly higher number of the infecting strains (sepsis and catheter related infections) of *S. epidermidis* possessed the *ica* and *mec*A genes as compared to the contaminating strains. On the contrary, our study was neither related to a single pathogen like *S epidermidis*, nor did we investigate to look for any molecular markers of pathogenicity for the studied pathogens. Despite that, our observations on the behaviour of fungal and bacterial biofilms in disease pathogenesis and antimicrobial resistance were the first of its kind from cases with orbital cellulitis and/or CRS, suggesting that biofilm formation could contribute to the chronicity *of* deep seated infections located in the orbit and paranasal sinuses and could help the organisms circumvent antimicrobial pressure and defence mechanism in the host.

## Conclusions

The recent discovery of biofilm formation in bacteria and yeasts led to a better understanding of microbial ecology and focussed new insights into the mechanisms of virulence and persistence of these pathogens. However, of late, it was generally assumed that filamentous fungi, some of which have a significant impact on our health and economy, were incapable of forming biofilms. In contrast to this assumption, we showed that surface-associated filamentous fungi could form biofilms. Based on these findings and on previous models utilising bacterial and yeast systems, we propose our preliminary hypothesis that filamentous fungi were able to form biofilms that could behave in a way much similar to that exhibited by bacteria in perpetuating antimicrobial resistance in a clinical setting. Future studies on the possibilities of developing modalities to dissolve the biofilms in vivo, would probably add further weapon to our arsenal in combating infections caused by these dangerous pathogens.
